# A multi‐modality physical phantom for mimicking tumor heterogeneity patterns in PET/CT and PET/MRI

**DOI:** 10.1002/mp.15853

**Published:** 2022-07-25

**Authors:** Alejandra Valladares, Thomas Beyer, Laszlo Papp, Elisabeth Salomon, Ivo Rausch

**Affiliations:** ^1^ QIMP Team, Centre for Medical Physics and Biomedical Engineering Medical University of Vienna Vienna Austria; ^2^ Centre for Medical Physics and Biomedical Engineering Medical University of Vienna Vienna Austria

**Keywords:** multi‐modality imaging, physical phantom, radiomics, tumor heterogeneity

## Abstract

**Background:**

Hybrid imaging (e.g., positron emission tomography [PET]/computed tomography [CT], PET/magnetic resonance imaging [MRI]) helps one to visualize and quantify morphological and physiological tumor characteristics in a single study. The noninvasive characterization of tumor heterogeneity is essential for grading, treatment planning, and following‐up oncological patients. However, conventional (CONV) image‐based parameters, such as tumor diameter, tumor volume, and radiotracer activity uptake, are insufficient to describe tumor heterogeneities. Here, radiomics shows promise for a better characterization of tumors. Nevertheless, the validation of such methods demands imaging objects capable of reflecting heterogeneities in multi‐modality imaging. We propose a phantom to simulate tumor heterogeneity repeatably in PET, CT, and MRI.

**Methods:**

The phantom consists of three 50‐ml plastic tubes filled partially with acrylic spheres of S1: 1.6 mm, S2: 50%(1.6 mm)/50%(6.3 mm), or S3: 6.3‐mm diameter. The spheres were fixed to the bottom of each tube by a plastic grid, yielding one sphere free homogeneous region and one heterogeneous (S1, S2, or S3) region per tube. A 3‐tube phantom and its replica were filled with a fluorodeoxyglucose (18F) solution for test–retest measurements in a PET/CT Siemens TPTV and a PET/MR Siemens Biograph mMR system. A number of 42 radiomic features (10 first order and 32 texture features) were calculated for each phantom region and imaging modality. Radiomic features stability was evaluated through coefficients of variation (COV) across phantoms and scans for PET, CT, and MRI. Further, the Wilcoxon test was used to assess the capability of stable features to discriminate the simulated phantom regions.

**Results:**

The different patterns (S1–S3) did present visible heterogeneity in all imaging modalities. However, only for CT and MRI, a clear visual difference was present between the different patterns. Across all phantom regions in PET, CT, and MR images, 10, 16, and 21 features out of 42 evaluated features in total had a COV of 10% or less. In particular, CONV, histogram, and gray‐level run length matrix features showed high repeatability for all the phantom regions and imaging modalities. Several of repeatable texture features allowed the image‐based discrimination of the different phantom regions (*p* < 0.05). However, depending on the feature, different pattern discrimination capabilities were found for the different imaging modalities.

**Conclusion:**

The proposed phantom appears suitable for simulating heterogeneities in PET, CT, and MRI. We demonstrate that it is possible to select radiomic features for the readout of the phantom. Most of these features had been shown to be relevant in previous clinical studies.

**Abbreviations**
CONV: conventional
COV: coefficient of variation
CT: computed tomography
GLCM: gray‐level co‐occurrence matrix
GLNUGLRLM: gray‐level nonuniformity for run
GLRLM: gray‐level run length matrix
GLZLM: gray‐level zone length matrix
HGRE: high gray‐level run emphasis
HGZE: high gray‐level zone emphasis
HU: Hounsfield units
LGRE: low gray‐level run emphasis
LGZE: low gray‐level zone emphasis
LRE: long‐run emphasis
LRHGE: long‐run high gray‐level emphasis
MRI: magnetic resonance imaging
PET: positron emission tomography
RLNU: run length nonuniformity
RP: run percentage
SD: standard deviation
SRE: short‐run emphasis
SRHGE: short‐run high gray‐level emphasis
SRLGE: short‐run low gray‐level emphasis
SUV: standardized uptake values
SZE: short‐zone emphasis
SZHGE: short‐zone high gray‐level emphasis
VOI: volume‐of‐interest

## 1 INTRODUCTION

Tomographic imaging, such as computed tomography (CT), magnetic resonance imaging (MRI), and positron emission tomography (PET), are used for the noninvasive characterization of oncological diseases. These systems are widely used in clinical routine for diagnosis and follow‐up examinations, through the visual assessment of the images and standard measures, such as tumor size, Hounsfield units (HU), apparent diffusion coefficient, and standardized uptake values (SUV).^1^, ^2^, ^3^, ^4^ However, these simple measures fall short of the ability to describe more complex patterns, such as intratumoral heterogeneities, that are often disease‐specific, and thus crucial for a comprehensive diagnosis.^5^, ^6^

Lately, radiomic features, in combination with artificial intelligence techniques, have been widely studied as an advanced tool for characterizing lesion heterogeneities. These approaches have proven advantageous in CT, MRI, and PET applications, for improved patient prognosis, staging, and predicting patient survival and recurrence of the disease.^7^, ^8^, ^9^, ^10^ Moreover, the combination of radiomic information from anatomical and functional imaging modalities, such as gained by PET/CT and PET/MR, has shown promising results toward advanced disease characterization and improved patient management.^11^, ^12^, ^13^

Nonetheless, radiomic features are more complex than standard measures, and their values are strongly affected by variations in acquisition protocols, post‐processing steps, and feature extraction methods.^14^, ^15^, ^16^, ^17^, ^18^, ^19^ Therefore, results from individual studies are rarely comparable, challenging the generalization of findings and the wider implementation of these approaches.^20^, ^21^, ^22^, ^23^ Related intra‐ and inter‐site variations in radiomic analysis can be addressed through phantom studies.^24^ However, developing phantoms to reproduce tumor heterogeneity for radiomic research is a challenge, particularly for multi‐modality imaging.

In contrast, the simulation of tumor heterogeneities for stand‐alone CT imaging is straightforward because of the wide range of suitable phantom materials.^25^ Typically, solids are used because of their stable temporal properties and ease of use, involving simple manufacturing processes and handling, making them suitable for multicenter studies and as reference objects.^18^, ^26^ However, most solid phantoms are not visible in standard MRI sequences. Visibility in PET images would require integrating long‐lived positron emitters, such as 68Ga/68Ge, resulting in high production costs and storage, handling, and transportation restrictions.

In the case of MRI, phantoms simulating heterogeneous patterns have been built using different materials (e.g., porous foams or polystyrene spheres) embedded in agarose solutions.^27^, ^28^, ^29^, ^30^, ^31^ Although suitable for MR imaging in a single‐center study, such approaches are difficult to extend to multicenter trials mainly due to the specific storage conditions required for the stability of the agar solutions. Furthermore, their use in PET is limited as the half‐life of standard PET isotopes (e.g., 18F or 68Ga) is short compared to the required phantom preparation times (e.g., stabilization of the agar gels).

In PET imaging, compartments filled with different isotope concentrations have been used to assess feature variations. Such phantom types are, in principle, suitable also for multicenter studies.^14^, ^19^, ^32^, ^33^, ^34^ However, the filling requires practical experience and preparing different radioactive stock solutions that may hamper reproducibility in practice. Further, to use them for CT or MRI would require the additional use of respective contrast agents in different concentrations, which results in highly complex preparation procedures.

In short, a couple of heterogeneity phantoms exist for the individual imaging modalities. However, to date, there is no report on their applicability in the context of cross‐modality imaging, such as with PET/CT and PET/MRI.^25^ Here, we propose a simple phantom concept to simulate heterogeneities in PET/CT and PET/MRI, which does not require the preparation of multiple activity/contrast agent concentrations and the filling of various compartments in a single phantom.

## 2 MATERIALS AND METHODS

### 2.1 Phantom

**FIGURE 1:**
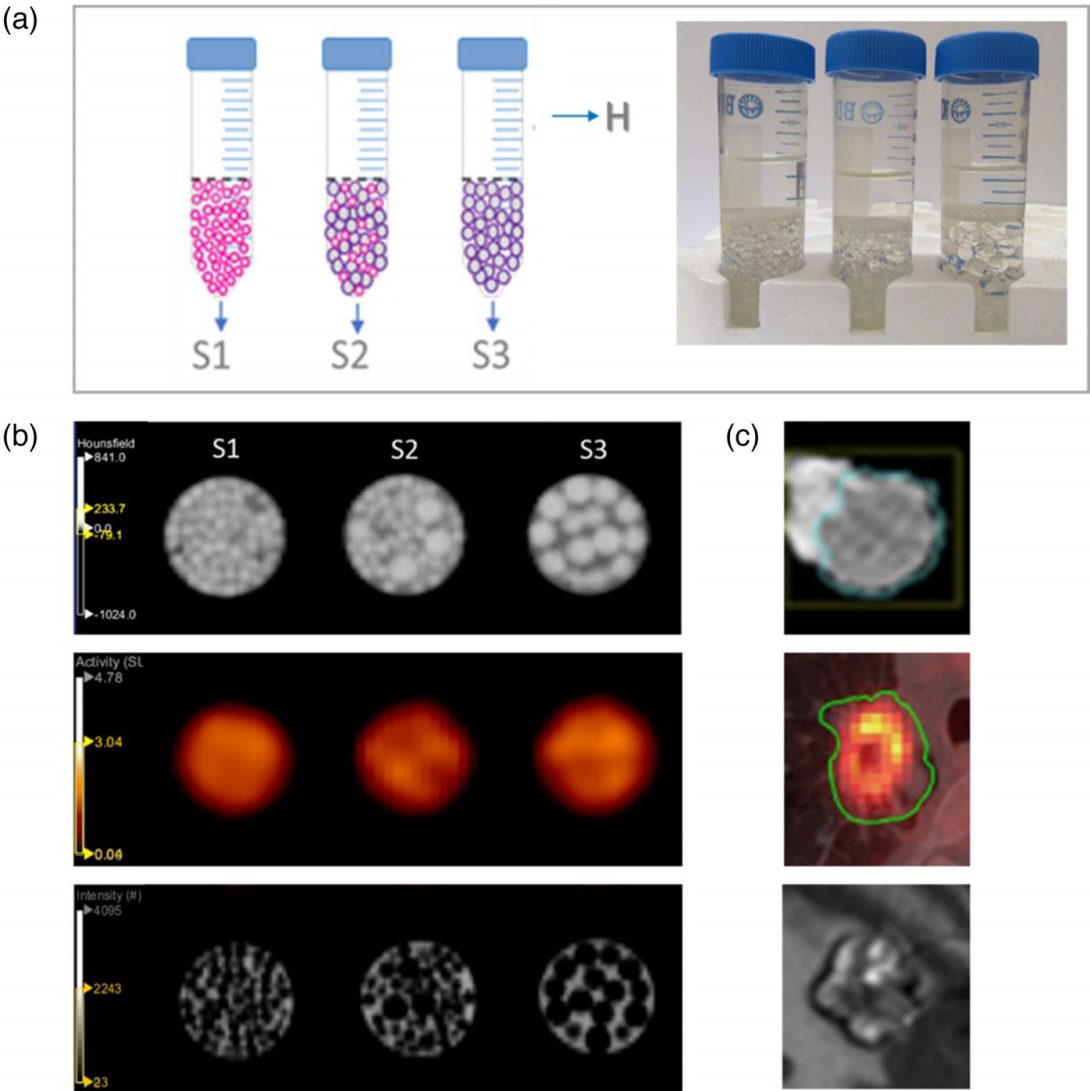
(a) A 3‐tube phantom filled partially with S1: 1.6‐mm diameter spheres, S2: 50% each of 1.6 and 6.3 mm, and S3: 6.3‐mm diameter spheres. H represents the homogeneous region. (b) From top to bottom: computed tomography (CT), positron emission tomography (PET), and magnetic resonance (MR) images of the phantom. (c) Examples of cancers that are represented with the proposed model; images adapted from previous reports^35^, ^36^, ^37^

The phantom comprises three conical tubes. Each tube consists of a homogeneous region containing only the radioactive solution (H) and a heterogeneous region established by acrylic spheres surrounded by a radioactive solution (S). For this, the tubes (*d* = 31 mm, *h* = 110 mm) were half‐filled with different sizes of acrylic spheres to recreate three different patterns (S1: 1.6 mm; S2: 50% each of 1.6 and 6.3 mm, and S3: 6.3‐mm diameter); see Figure 1. The homogeneous area was separated from the sphere area using a 3D‐printed plastic grid. All tubes were filled with a fluorodeoxyglucose (18F) aqueous solution with 20‐kBq/ml activity concentration at the PET acquisition start time.

### 2.2 Measurements

We built the 3‐tube phantom twice (P1, P2) and evaluated differences in the radiomic features in a test–retest scenario to test the radiomic features’ repeatability for our proposed phantom concept. Two consecutive scans, with physical repositioning of the phantom between them, were performed on a Biograph TPTV PET/CT system (Siemens Healthineers, Germany) and a Siemens Biograph mMR PET/MR system for PET/CT and MRI measurements, respectively. Both of the phantoms were centered in the field of view of the systems. For the PET and CT measurements, a standard PET/CT oncological protocol was used.

Specifically, the PET measurements were performed for 10‐min acquisition time and a single‐bed position; reconstructed using CT‐based attenuation and scatter correction, matrix size of 336 × 336, voxel size of ∼1.0 × 1.0 × 1.0 mm^3^, and a 5‐mm Gaussian filter. The CT images were acquired at 120 kVp, 152 mAs with a slice thickness of 1 mm, matrix size of 512 × 512 pixels, and voxel size equal to 0.6 × 0.6 × 1.0 mm^3^. MRI scans were performed using a body coil. T1‐weighted MR images were acquired using an inversion recovery sequence with a matrix size = 256 × 256, repetition time = 1500 ms, echo time = 2 ms, TI = 900 ms, number of averages = 1, pixel bandwidth = 250 Hz/px, flip angle = 8°, field of view = 262 × 262 mm^2^, slice thickness = 1 mm, and no interslice gap. T2‐weighted MR images were acquired with turbo spin‐echo sequence, matrix size = 256 × 208, repetition time = 9630 ms, echo time = 92 ms, number of averages = 1, pixel bandwidth = 200 Hz/px, flip angle = 120°, field of view = 173 × 214 mm^2^, slice thickness = 0.8 mm, and 1.2‐mm interslice gap. All images were stored in 16‐bit DICOM format.

### 2.3 Feature extraction

Spherical volumes‐of‐interests (VOI) of 4.6 ml were placed centrally in the homogeneous and heterogeneous regions of each PET, CT, and MR image volume. Conventional (CONV) measures such as SUVmean and average CT‐HU were extracted from volumes of interest placed on PET and CT images, respectively. Furthermore, from all phantom regions and imaging modalities, 42 radiomic features (10 first order and 32 texture features) were calculated using the open‐source software LIFEx.^38^ The same VOIs shape and size were used. A fixed bin width and 2‐mm spatial resampling was applied for all modalities. The parameters for feature extraction were selected based on previous recommendations from patient and phantom studies.^15^, ^36^, ^39^ Matrices and extracted features are listed in Table S1.

### 2.4 Statistical analysis

#### 2.4.1 Repeatability of radiomic features

We used the coefficient of variation (COV), which is the ratio of the standard deviation (SD) to the mean (Equation 1), expressed as a percentage to assess the repeatability of the radiomic features for each imaging modality:

(1) $$\mathrm{COV}=\frac{\;\sigma \;}{\mu }$$

As test–retest measurements are supposed to measure the same parameter over time, we calculated a single COV by pooling the data across phantoms and scans. We considered a COV of 10% or less an indicator of high repeatability of a specific radiomic feature and high reproducibility of the phantom itself.^15^

#### 2.4.2 Pattern discrimination

For each imaging modality, we used Wilcoxon's tests to evaluate the ability of the radiomic features to (1) separate homogeneous from heterogeneous regions and (2) discriminate among the three heterogeneous patterns (S1, S2, and S3). We selected the repeatable features (COV < 10%) and compared their values for pairs of phantom regions. We ran the test at a 5% level of significance. In this part of the analysis, we considered only textural features.

Different characteristics of a lesion are described through PET, CT, and MRI; therefore, we analyzed each imaging modality separately. Individual feature values from each modality were normalized to the average over the homogeneous regions from the two phantoms and scans. Normalized feature values were represented as Boxplots for the H region and individual points for the S regions.

## 3 RESULTS

### 3.1 Conventional measures and visual assessment

The average SUVmean (±SD) for the homogeneous regions across the phantoms and scans was 4.5 (±0.1), whereas for the heterogeneous regions, SUVmean for Phantom 1 were 1.7 (0.1), 1.6 (0.1), and 1.8 (0.1) for S1, S2, and S3, respectively. Similar values were obtained for Phantom 2 and test–retest, as shown in Table 1. SUVmean in all the heterogeneous regions was decreased by a factor of 3 compared to the homogeneous region by replacing the activity with the acrylic spheres. Mean CT‐HU across the homogeneous regions was 11 HU and varied between 78 and 88 HU for the heterogeneous regions (Table 1). Unlike in PET images, the simulated texture patterns were easily distinguished by the human eye in CT and MR images (Figure 1).

**TABLE 1:** SUVmean (±SD) and HU values (±SD) across phantoms (P1 and P2) and test–retest scans

Modality	Scan	Parameter	Homogeneous region (H)	Phantom 1 (P1)			Phantom 2 (P2)		
				S1	S2	S3	S1	S2	S3
PET	Test	SUVmean	4.5 (0.1)	1.7 (0.1)	1.6 (0.1)	1.8 (0.1)	1.7 (0.1)	1.5 (0.1)	1.8 (0.1)
	Retest	SUVmean	4.5 (0.1)	1.7 (0.1)	1.6 (0.1)	1.8 (0.1)	1.7 (0.1)	1.5 (0.1)	1.8 (0.1)
CT	Test	HU	11.1 (3.8)	83.3 (21.7)	85.1 (28.1)	78.6 (42.1)	87.3 (23.9)	85.5 (27.3)	80.2 (42.3)
	Retest	HU	11.1 (3.5)	82.8 (24.5)	84.4 (28.2)	77.9 (42.2)	87.8 (24.2)	87.7 (26.2)	79.9 (42.9)

Abbreviations: CT, computed tomography; HU, Hounsfield units; PET, positron emission tomography.

### 3.2 Repeatability of radiomic features

**FIGURE 2:**
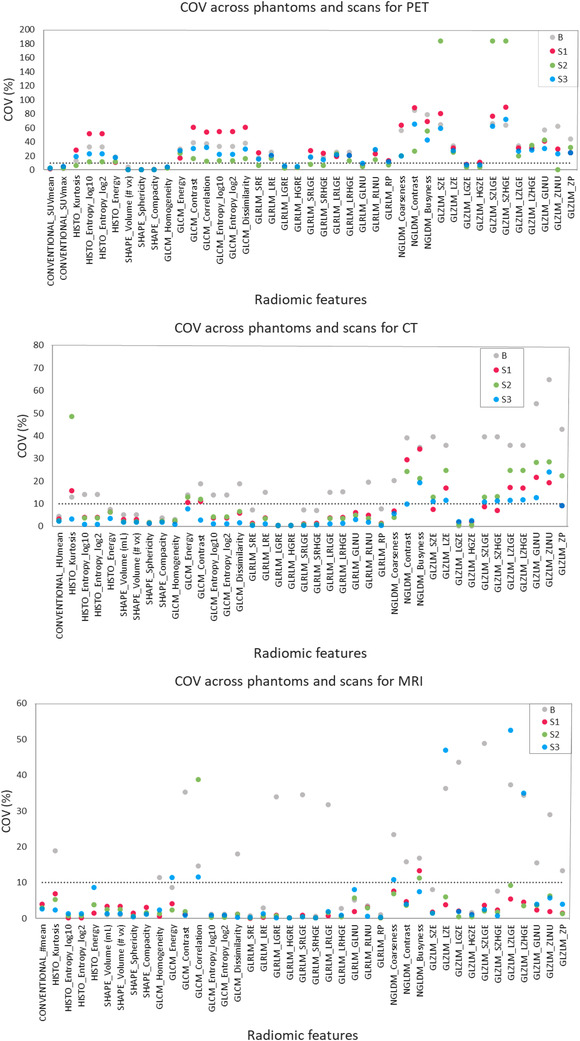
Coefficient of variation (COV) (%) per radiomic feature across phantoms and scans for positron emission tomography (PET) (top), computed tomography (CT) (middle), and magnetic resonance imaging (MRI) (bottom). Dashed lines indicate COV ≤ 10%.

Of the 42 evaluated features in PET, CT, and MRI, 10, 16, and 21 presented with a COV ≤ 10%, respectively, for all phantom regions (Figure 2). CONV, histogram, and gray‐level run length matrix features showed high repeatability for all the phantom regions and imaging modalities. GLCM (gray‐level co‐occurrence matrix) features from CT and MRI also had low COVs, especially for the three S regions. GLZLM (gray‐level zone length matrix) features had low COVs for all phantom regions only in MRI. Overall, MR images had the highest number of stable radiomic features for the proposed phantom.

Further, the number of repeatable features differed across phantom regions, without a specific tendency related to the different sphere sizes. Only for PET images, S2 presented a considerably higher number of repeatable features (COV ≤ 10%) than the other phantom regions.

### 3.3 Pattern discrimination

**FIGURE 3:**
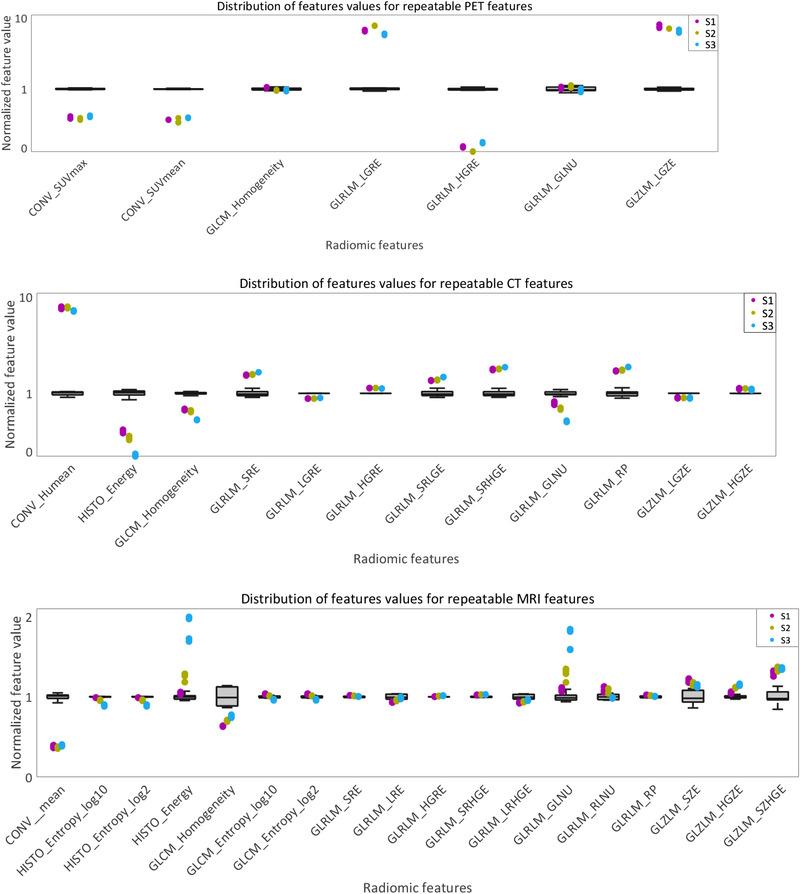
Feature values for positron emission tomography (PET) (top), computed tomography (CT) (middle), and magnetic resonance imaging (MRI) (bottom). The figure only includes those features with coefficient of variation (COV) ≤10%. Boxplots indicate the distribution of the values for homogeneous regions across phantoms and scans. The individual values (*n* = 4 from test/retest from both replicates) for S1, S2, and S3 are superposed on each boxplot.

Figure 3 presents the distribution of the normalized values for features with COV ≤ 10% in each imaging modality. Tables 2, 3, 4 contain Wilcoxon's test results applied among paired phantom regions in PET, CT, and MR images. Most of the PET‐based radiomic features that presented a COV < 10% in the test–retest scans distinguished S1, S2, and S3 (*p* = 0.029) and discriminated them from the homogeneous region (*p* = 0.001). For CT, only two features were significantly different for all the paired regions. Most of the other CT radiomic features distinguished S from H regions (*p* = 0.01) and S3 from S1 and S2 (*p* = 0.029). No significant difference was found between S1 and S2 (*p* > 0.05). Six of the MRI features presented significant differences among all the phantom regions. Discrimination of S1, S2, and S3 was variable across the rest of MRI‐based features.

**TABLE 2:** *p*‐Values of Wilcoxon's test for positron emission tomography (PET) texture indices among paired phantom regions

Feature matrix	Feature name	S1 vs. H	S2 vs. H	S3 vs. H	S1 vs. S2	S1 vs. S3	S2 vs. S3
Conventional	SUVmean^a^	0.001	0.001	0.001	0.029	0.029	0.029
	SUVmax^a^	0.001	0.001	0.001	0.200	0.114	0.029
GLCM	Homogeneity^a^	0.042	0.030	0.316	0.029	0.029	0.486
GLRLM	LGRE^a^	0.001	0.001	0.001	0.029	0.029	0.029
	HGRE^a^	0.001	0.001	0.001	0.029	0.029	0.029
	GLNU^a^	0.379	0.058	0.521	0.114	0.114	0.029
GLZLM	LGZE^a^	0.001	0.001	0.001	0.029	0.029	0.029

*Note*: “H” corresponds to the homogeneous region. Gray‐colored cells correspond to *p* > 0.05, no significant difference.

Abbreviations: GLCM, gray‐level co‐occurrence matrix; GLNU, gray‐level nonuniformity; GLRLM, gray‐level run length matrix; GLZLM, gray‐level zone length matrix; HGRE, high gray‐level run emphasis; LGRE, low gray‐level run emphasis; LGZE, low gray‐level zone emphasis.

^a^ PET features previously reported on clinical trials as robust to the number of gray levels for intensity discretization, suggested for future studies on tumor response characterization or showing some reliability to build multi‐parametric models.^41^, ^42^, ^43^

**TABLE 3:** *p*‐Values of Wilcoxon's test for computed tomography (CT) texture indices among paired phantom regions

Feature matrix	Feature name	S1 vs. H	S2 vs. H	S3 vs. H	S1 vs. S2	S1 vs. S3	S2 vs. S3
Histogram	Energy	0.001	0.001	0.001	0.029	0.029	0.029
GLCM	Homogeneity^a^	0.001	0.001	0.001	0.057	0.029	0.029
GLRLM	SRE	0.001	0.001	0.001	0.114	0.029	0.029
	LGRE	0.001	0.001	0.001	0.829	0.029	0.029
	HGRE^a^	0.001	0.001	0.001	0.686	0.029	0.029
	SRLGE	0.001	0.001	0.001	0.114	0.029	0.029
	SRHGE	0.001	0.001	0.001	0.114	0.029	0.029
	GLNU^a^	0.001	0.001	0.001	0.029	0.029	0.029
	RP	0.001	0.001	0.001	0.057	0.029	0.029
GLZLM	LGZE^a^	0.001	0.001	0.001	1	0.971	0.286
	HGZE^a^	0.001	0.001	0.001	1	0.343	0.029

*Note*: “H” corresponds to the homogeneous region. Gray‐colored cells correspond to *p* > 0.05.

Abbreviations: GLCM, gray‐level co‐occurrence matrix; GLNU, gray‐level nonuniformity; GLRLM, gray‐level run length matrix; GLZLM, gray‐level zone length matrix; HGRE, high gray‐level run emphasis; HGZE, high gray‐level zone emphasis; LGRE, low gray‐level run emphasis; LGZE, low gray‐level zone emphasis; RP, run percentage; SRE, short‐run emphasis; SRLGE, short‐run low gray‐level emphasis; SRHGE, short‐run high gray‐level emphasis.

^a^ CT features previously reported on clinical trials as reproducible radiomic features under a wide range of imaging parameter settings or potentially reliable to build prognosis models.^42^, ^44^, ^45^ ^.^

**TABLE 4:** *p*‐Values of Wilcoxon's test for magnetic resonance imaging (MRI) texture indices among paired phantom regions

Feature matrix	Feature name	S1 vs. H	S2 vs. H	S3 vs. H	S1 vs. S2	S1 vs. S3	S2 vs. S3
Histogram	Entropy_log10	0.001	0.001	0.001	0.029	0.029	0.029
	Entropy_log2	0.001	0.001	0.001	0.029	0.029	0.029
	Energy^a^	0.054	0.001	0.001	0.029	0.029	0.029
GLCM	Homogeneity^a^	0.001	0.001	0.001	0.029	0.029	0.029
	Entropy_log10^a^	0.001	0.098	0.001	0.029	0.029	0.029
	Entropy_log2	0.001	0.098	0.001	0.029	0.029	0.029
GLRLM	SRE	0.001	0.001	0.751	0.029	0.029	0.029
	LRE	0.001	0.001	0.663	0.029	0.029	0.029
	HGRE^a^	0.019	0.001	0.001	0.029	0.029	0.029
	SRHGE^a^	0.001	0.001	0.001	0.029	0.029	0.886
	LRHGE^a^	0.001	0.001	0.002	0.057	0.029	0.029
	GLNU^a^	0.012	0.001	0.001	0.029	0.029	0.029
	RLNU^a^	0.001	0.004	0.841	0.343	0.029	0.029
	RP	0.001	0.001	0.751	0.029	0.029	0.029
GLZLM	SZE^a^	0.001	0.001	0.001	0.057	0.029	0.343
	HGZE^a^	0.001	0.001	0.001	0.029	0.029	0.029
	SZHGE^a^	0.001	0.001	0.001	0.114	0.029	0.886

*Note*: “H” corresponds to the homogeneous region. Gray‐colored cells correspond to *p* > 0.05.

Abbreviations: GLCM, gray‐level co‐occurrence matrix; GLNU, gray‐level nonuniformity; GLRLM, gray‐level run length matrix; GLZLM, gray‐level zone length matrix; HGRE, high gray‐level run emphasis; HGZE, high gray‐level zone emphasis; LRE, long‐run emphasis; LRHGE, long‐run high gray‐level emphasis; RLNU, run length nonuniformity; RP, run percentage; SRE, short‐run emphasis; SRHGE, short‐run high gray‐level emphasis; SZE, short‐zone emphasis; SZHGE, short‐zone high gray‐level emphasis.

^a^ Features reported as robust to segmentation methods or helpful on building prognosis models in previous clinical MRI studies.^43^, ^46^, ^47^, ^48^

## 4 DISCUSSION

We developed a simple phantom for simulating different textures in dual‐modality images involving PET, CT, and MRI (Figure 1). The phantom consisted of three plastic tubes filled with acrylic spheres embedded in a radioactive solution. By varying the acrylic sphere sizes, we generated three different image patterns (S1–S3). Two phantoms were built and measured in a test–retest scenario. Specific radiomic features yielded low inter‐phantom and inter‐scan variability and good capability to distinguish among phantom regions, thus supporting the ability of the proposed phantom design to mimic heterogeneities in PET, CT, and MRI or combinations thereof.

The intent to build a phantom suitable for PET, CT, and MR imaging rests upon a practical issue. Scientific radiomic studies in nuclear medicine imaging usually suffer from a very small number of datasets,^55^, ^56^ limiting the quality of these studies and their clinical relevance. Data pooling can benefit from harmonizing imaging studies and the standardization of imaging readouts for radiomic studies. Imaging can be harmonized through phantom studies by the on‐site physicist or the technologist's team. The phantom concept proposed here is a simple and easy‐to‐adopt approach to assessing heterogeneity in multi‐modality imaging. Compared to existing multi‐modality phantoms,^25^ our model does not require filling multiple compartments to create homogeneous and heterogeneous patterns in PET/MRI and PET/CT, which helps to reduce the effect of variation in phantom preparation on the result of harmonization efforts. Moreover, the filling/refilling of the phantom is achieved easily with a long needle syringe. It also does not require specific conditions for storage, for example, temperature, and humidity, which is beneficial for long‐term and multicenter studies.

For the used phantom design in this study, we found a set of repeatable radiomic features (COV ≤ 10%) across the two phantoms and scans for each imaging modality. However, as observed in Figure 2, the repeatability of those features was variable across the phantom regions. In general, features from heterogeneous regions were somewhat more stable than those from homogeneous regions. This is in‐line with a previous study reporting the dependence of PET‐based features repeatability on the recreated heterogeneities, object sizes, and uptake ratios; the authors suggested that noise reduction, for example, by image smoothing, may lead to higher repeatability for homogeneous regions.^49^ However, in our study, no image noise reduction was applied to avoid a potential loss of textural information from the heterogeneous regions. The application of noise reduction methods needs to be further addressed for radiomic analysis in multi‐modality imaging due to the varying noise sources across PET, CT, and MRI.^50^ Overall, texture features were more variable than first‐order features (e.g., SUV, HU, and histogram). This can be explained to some extent by the effects of rebinning and rescaling parameters used during the extraction of the features on the repeatability of texture indexes, as reported in phantom and patient radiomic studies in multiple imaging modalities.^17^

Most of the repeatable radiomic features (COV < 10%) obtained for the proposed phantom were able to discriminate homogeneous and heterogeneous patterns (*p* = 0.01) while presenting smaller differences among heterogeneous patterns (Tables 2, 3, 4). One reason is that there are no subtle heterogeneity differences among the simulated patterns besides the diverse sphere sizes. Imaging objects showing a wider range of SUV values, CT, and MR contrasts may result in larger heterogeneity and better discrimination by radiomic texture indexes.^30^, ^51^, ^52^ However, already this simple concept is able to mimic specific cancer types (Figure 1).

We expected that the arrangement of different sphere sizes in the S2 would lead to a more heterogeneous pattern than the ones created with single sphere sizes in S1 and S3 regions, which can be seen as the same pattern but at different scales. Thus, the ability to discriminate S2 from S1 and S3 was expected to be higher than between S1 and S3. Nonetheless, this assumption was not supported by the results. Instead, the variability of pattern discrimination across the S regions in the three imaging modalities supports that subtle heterogeneity differences and scaling of the same heterogeneous object influence pattern discrimination through radiomic features.^49^ The differences seen between S1 and S3 might be caused by partial volume effects, which have a stronger impact on smaller objects and the different sizes of connected homogeneous regions when using different sphere sizes in combination with fixed voxel dimensions. Further, the pattern S2 is a mixture of the patterns in S1 and S3, and thus, it seems reasonable that feature values extracted from S2 are in a similar range as feature values extracted from S1 and S3.

We also found that the same feature can present different pattern discrimination capabilities for the different imaging modalities. For example, unlike in PET, the gray‐level nonuniformity feature from CT and MRI was significantly different across all the paired regions. Moreover, more MRI features helped discriminate among the simulated patterns compared to features extracted from PET and CT. These results may be linked to the differences in the spatial resolution of the systems and the phantom composition. First, images with high spatial resolution, as is the case for MRI and CT, allow a better classification of texture patterns.^30^ Second, our phantom was composed only of acrylic spheres and water, thus, yielding a higher image contrast in MR than in PET and CT. It indicates a need to consider the appearance of the material under each imaging modality during the phantom design to produce further enhanced heterogeneous patterns in different imaging systems.

PET images of the three recreated patterns presented a similar visual appearance attributed to the relatively high noise level and a low spatial resolution of the PET system compared to CT and MRI (Figure 1b). Nevertheless, some stable PET radiomic features had good discrimination capability (*p* < 0.05) among all the phantom regions. This outcome supports the statement that radiomic features may describe patterns in images that are not visible to the human eye.^24^

It is worth noting that some of the features that performed well in discriminating the phantom regions have been reported to help in differentiating homogeneous from heterogeneous body tissues and classifying heterogeneous lesions in patient studies (Tables 2, 3, 4). For example, Orlhac et al.^41^ reported SUV_max_, homogeneity, and low gray‐level zone emphasis PET features obtained by absolute resampling to be significantly different between tumor and healthy tissue in non‐small cell lung cancer patients. These features were also helpful to discriminate between adenocarcinoma and squamous cell carcinoma. Likewise, in a similar study, the values for those features presented significant differences between homogeneous and heterogeneous breast lesions.^54^ In an MRI patient study, the authors found, for example, energy and entropy helpful within a machine learning–based classification system for providing a prediction of the methylation status of a strong predictive marker for therapy success in brain tumors, specifically glioblastomas.^48^

There are still some limitations with the proposed model; for example, to improve further usability in multicenter trials, the phantom could be modified to fit existing standardized imaging phantoms, such as the National Electrical Manufacturers Association IQ phantom. 3D printing could be used to make the phantom compatible with such standardized image quality phantoms or optimize the phantom (e.g., other geometries) to be also helpful to evaluate shape features. We also suggest exploring MRI signal generating materials for future studies.^25^ In addition, future studies need to systematically determine an appropriate compromise among the spatial resolutions of the imaging systems, type of recreated patterns, and the used phantom materials. This needs to be done in view of the different clinical scenarios to provide results as close as possible to those obtained from patient studies due to the differences seen in feature stabilities between the different heterogeneities and imaging methods.

For the present study, all acquisitions were performed on a 3T MRI as part of commonly used hybrid imaging systems in nuclear medicine. However, the field strength influences achievable resolution and noise properties; thus, we expect variations for specific features across MRI systems,^53^ which would need to be addressed in future studies for a broad implementation of MRI‐based radiomic studies across centers.

## 5 CONCLUSION

We propose a simple phantom consisting of acrylic spheres embedded in a radioactive solution to simulate tumor heterogeneities in PET, CT, MRI studies, or combinations thereof. We demonstrated that it is possible to select radiomic features relevant in previous clinical studies for the readout of the phantom.

## CONFLICT OF INTEREST

The authors declare that the research was conducted without any commercial or financial relationships that could be construed as a potential conflict of interest.

## Supporting information


**Table S1** Calculated matrices and corresponding radiomic features
