# Supplementary material for: A multi‐modality physical phantom for mimicking tumor heterogeneity patterns in PET/CT and PET/MRI
Source: Med Phys. 2022 Jul 25;49(9):5819–29. doi: 10.1002/mp.15853 (PMC9543355; doi:10.1002/mp.15853)
Supplement: Supplementary file 1 — Table S1 Calculated matrices and corresponding radiomic features [file MP-49-5819-s001.docx]

**Supplemental data**

**Supplemental Table 1.** Calculated matrices and corresponding radiomic features.

| **Texture features** | GLCM (Grey-level co-occurrence matrix) | Homogeneity (=Inverse difference), Energy (=Uniformity or Angular second moment), Contrast (=Variance or Inertia), Correlation, Entropy_log10, Entropy_log2 (=Joint entropy), Dissimilarity |
| --- | --- | --- |
|  | NGLDM (neighbourhood grey-level different matrix) | Coarseness, contrast, busyness |
|  | GLRLM (grey-level run length matrix) | Short-Run Emphasis (SRE), Long-Run Emphasis (LRE), Low Gray-level Run Emphasis (LGRE), High Gray-level Run Emphasis (HGRE), Short-Run Low Gray-level Emphasis (SRLGE), Short-Run High Gray-level Emphasis (SRHGE), Long-Run Low Gray-level Emphasis (LRLGE), Long-Run High Gray-level Emphasis (LRHGE), Gray-Level Non-Uniformity for run (GLNU), Run Length Non-Uniformity (RLNU), Run Percentage (RP) |
|  | GLZLM  (or GLSZM, grey-level zone length matrix) | Short-Zone Emphasis (SZE), Long-Zone Emphasis (LZE), Low Gray-level Zone Emphasis (LGZE), High Gray-level Zone Emphasis (HGZE), Short-Zone Low Gray-level Emphasis (SZLGE), Short-Zone High Gray-level Emphasis (SZHGE), Long-Zone Low Gray-level Emphasis (LZLGE) Long-Zone High Gray-level Emphasis (LZHGE), Gray-Level Non-Uniformity for zone (GLNU), Zone Length Non-Uniformity (ZLNU), Zone percentage (ZP) |
| **First-order features** | Conventional | Mean |
|  | Histogram | Skewness, Kurtosis, Entropy, Entropy_log2, Energy (=Uniformity) |
|  | Shape | Sphericity, Compacity, Volume |
